# Optimized cell growth and poly(3-hydroxybutyrate) synthesis from saponified spent coffee grounds oil

**DOI:** 10.1007/s00253-022-12093-9

**Published:** 2022-08-27

**Authors:** Haydn Rhys Ingram, Risto John Martin, James Benjamin Winterburn

**Affiliations:** 1 Department of Chemical Engineering, The Mill, The University of Manchester, Manchester, M13 9PL UK; 2 Department of Engineering Science, Institute of Biomedical Engineering, University of Oxford, Oxford, OX3 7DQ UK

**Keywords:** Polyhydroxyalkanoates, *Cupriavidus necator*, Spent coffee grounds, Coffee oil, Saponification

## Abstract

**Abstract:**

Spent coffee ground (SCG) oil is an ideal substrate for the biosynthesis of polyhydroxyalkanoates (PHAs) by *Cupriavidus necator*. The immiscibility of lipids with water limits their bioavailability, but this can be resolved by saponifying the oil with potassium hydroxide to form water-soluble fatty acid potassium salts and glycerol. Total saponification was achieved with 0.5 mol/L of KOH at 50 °C for 90 min. The relationship between the initial carbon substrate concentration (*C*_0_) and the specific growth rate (*µ*) of *C. necator* DSM 545 was evaluated in shake flask cultivations; crude and saponified SCG oils were supplied at matching initial carbon concentrations (*C*_0_ = 2.9–23.0 g/L). The Han-Levenspiel model provided the closest fit to the experimental data and accurately described complete growth inhibition at 32.9 g/L (*C*_0_ = 19.1 g/L) saponified SCG oil. Peak *µ*-values of 0.139 h^−1^ and 0.145 h^−1^ were obtained with 11.99 g/L crude and 17.40 g/L saponified SCG oil, respectively. Further improvement to biomass production was achieved by mixing the crude and saponified substrates together in a carbon ratio of 75:25% (w/w), respectively. In bioreactors, *C. necator* initially grew faster on the mixed substrates (*µ* = 0.35 h^−1^) than on the crude SCG oil (*µ* = 0.23 h^−1^). After harvesting, cells grown on crude SCG oil obtained a total biomass concentration of 7.8 g/L and contained 77.8% (w/w) PHA, whereas cells grown on the mixed substrates produced 8.5 g/L of total biomass and accumulated 84.4% (w/w) of PHA.

**Key points:**

• *The bioavailability of plant oil substrates can be improved via saponification.*

• *Cell growth and inhibition were accurately described by the Han-Levenpsiel model.*

• *Mixing crude and saponified oils enable variation of free fatty acid content.
*

## Introduction

In the 2018/2019 crop year, around 20.26 million tonnes of green coffee were produced globally (International Coffee Organisation 2020). However, approximately 90% (dry w/w) of the whole coffee fruit is discarded as solid waste in the form of pulp, husk, silver skin, and spent coffee grounds (SCGs) (Murthy and Naidu 2012; Salazar-López et al. 2020). Coffee is predominantly either used to produce soluble (instant) coffee or directly brewed (boiled, filtered or high-pressured) into the coffee beverage (Mussatto et al. 2011; Gómez-de la Cruz et al. 2015). These processes generate vast volumes of SCGs as a by-product, which are of fine particle size, high humidity (50–85%, w/w), and organic load. Considering that around 650 kg of wet SCG are obtained from every tonne of green coffee (Murthy and Naidu 2012), approximately 13.2 million tonnes of wet SCG were produced from the coffee grown in the 2018/2019 crop year. Despite being of rich chemical composition with minimal contamination, the vast majority of SCG are discarded into landfills, incinerated, or used as animal feed (Machado et al. 2012).

SCGs consist predominantly of lignocellulose—a covalently bonded network of lignin (25–33%, w/w) and hemicellulose (30–40%) polymers surrounding a crystalline cellulose core (8.6–13.3%), as well as lipids (10–20%), proteins (6.7–13.6%), and phenolic compounds (2.5%) (Obruca et al. 2015). Owing to this, as well as their high availability and low cost, the utilization of SCGs has garnered increasing interest in recent years. SCGs could potentially be used in a wide range of applications, including directly as a fuel, as an adsorption material, and in the production of biodiesel, bioethanol, and other higher-value products, including polyhydroxyalkanoates (PHAs) (Karmee 2018; Kovalcik et al. 2018; McNutt and He 2019). The concept of an SCG-based biorefinery, in which feedstocks undergo a series of integrated processes to convert them into value-added products, has gathered significant attention in recent years (Aristizábal-Marulanda et al. 2017; Caetano et al. 2017; Kourmentza et al. 2018; Girotto et al. 2018; Mata et al. 2018; Karmee 2018; Kovalcik et al. 2018; Zabaniotou and Kamaterou 2019; Atabani et al. 2019; Massaya et al. 2019; Rajesh Banu et al. 2020; Battista et al. 2020).

PHAs are biodegradable, bio-based, and biocompatible polymers with similar physical properties to certain petroleum-derived plastics, such as polypropylene. PHAs are synthesized and accumulated by several bacteria and archaea as discrete granules in the cell cytoplasm. Organisms primarily use them as a form of carbon and energy storage (Bugnicourt et al. 2014), but their inclusion also protects the cells from environmental stress conditions, such as high temperatures, freezing, osmotic shock, UV irradiation, and desiccation (Obruca et al. 2018). The wide-scale use of PHAs is currently prevented by their relatively high production costs and poor material processing properties (Surendran et al. 2020; Ingram and Winterburn 2021). The issue of cost can be helped by utilizing cheap carbon substrates, such as waste plant oils. Recently, oil extracted from SCGs has been demonstrated to be a suitable feedstock for the microbial production of PHAs by *Cupriavidus necator* (Cruz et al. 2014; Obruca et al. 2014; Bhatia et al. 2018; Ingram and Winterburn 2021, 2022). However, adding a plant oil to an aqueous growth medium forms a heterogeneous mixture, in which the low-density oil floats on the surface (Budde et al. 2011). This phenomenon creates a bottleneck in the process by limiting substrate availability and product recovery. Also, the quantification of the various metabolites must be performed offline, involving the use of hazardous organic solvents in multiple time-consuming stages. Furthermore, plant oil substrates often lead to varying lag times and difficulties in taking early representative samples (Budde et al. 2011; Cruz 2015).

Plant oils and animal fats predominantly consist of acylglycerides, which are esters of 1 to 3 fatty acids attached to a glycerol backbone. The acylglycerides can be saponified via reactions with sodium or potassium hydroxide to form fatty acid salts and glycerol. Unlike acylglycerides, the products of saponification are relatively water-soluble (Rustan and Drevon 2005; Ferre-Guell and Winterburn 2019), and can be rapidly absorbed to be metabolized by microorganisms, such as *C. necator*, without the need for enzymatic pretreatment (Obruca et al. 2014). In this context, saponification has often been used as a pretreatment method for fermentations involving *Pseudomonads,* as some species lack the lipases necessary for enzymatic hydrolysis (Tan et al. 1997; Allen et al. 2010; Mozejko and Ciesielski 2013; Bustamante et al. 2019; Boonyawanich et al. 2021). The content of free fatty acids (FFAs) in plant oil substrates has been demonstrated to strongly influence the production of P(3HB) by *C. necator* (Obruca et al. 2014). FFAs are natural surfactants that emulsify acylglycerides, which renders them more susceptible to the action of lipases through the increased contact area, solubility, and bioavailability (Budde et al. 2011; Lu et al. 2013; Ferre-Guell and Winterburn 2019). Hence, saponification could be used as a potential means to increase cell growth rates and PHA productivity of *C. necator* by increasing the FFA content of plant oil substrates.

High substrate concentrations can inhibit the cell growth rate due to the influence of ionic strength, osmotic pressure, or overloading of the membrane transport systems (Blanch and Clark 1997). In addition, surfactants can act as antimicrobial agents through lysis and solubilization of the cellular membrane (i.e., lipid bilayer) (Nazari et al. 2012; Ferre-Guell and Winterburn 2019). Therefore, this study assesses the effects of saponification of the crude SCG oil on the ability of *C. necator* DSM 545 to use the substrate for biomass and PHA production. An evaluation of the effect of the initial carbon concentration (*C*_0_) on the organism’s specific growth rate, *μ*, was also carried out in shake flasks to optimize the product yields. These results were then compared to several widely published kinetic growth models, extended or evolved from the Monod model. Further cultivations were performed, in which crude and saponified SCG oils were mixed in a range of different carbon ratios to evaluate if the saponified oil could act as a consumable surfactant. Finally, fermentations were carried out in 3-L bioreactors operated in batch mode, and the overall performances were assessed and compared with similar cultivations reported in the literature.

## Materials and methods

### Materials and microorganism

SCG oil, extracted with subcritical water, was purchased from Merck (product W530639, Darmstadt, Germany). All the other chemicals and reagents used in this study were of analytical grade and obtained from either Merck (Darmstadt, Germany) or Thermo Fisher Scientific (Loughborough, UK). *C. necator* DSM 545 (H1G^+3^) was obtained from the Leibniz Institute DSMZ (Braunschweig, Germany) in freeze-dried form and reactivated as previously described (Ingram and Winterburn 2021).

### Saponification of SCG oil

Each gram of “crude” SCG oil was saponified with 10 mL of ethanolic KOH solution in 50 mL glass Duran™ bottles in a temperature-controlled incubator. A range of conditions was evaluated to optimize the process, including the concentration of KOH (0.50–1.00 mol/L), temperature (50–70 °C), and reaction time (30–90 min). Each sample in the optimization study used 2 g of SCG oil dissolved in 20 mL of ethanolic KOH solution. The resulting solutions contained a mixture of potassium salts of fatty acids, glycerol, and unsaponified substances, all dissolved in ethanol. The saponification value of each sample (SV_sample_) was directly measured and compared with that of the original oil sample (SV_oil_) to determine the extent of saponification, as per Eq. (1).

1 $$\mathrm{Extent}\;\mathrm{of}\;\mathrm{Saponification}\;(\boldsymbol\%)=\frac{{\mathrm{SV}}_{\mathrm{sample}}}{{\mathrm{SV}}_{\mathrm{oil}}}\times100$$

SV_oil_ was previously determined to be 175.7 ± 2.3 mg_KOH_ g_oil_^−1^ (Ingram and Winterburn 2022). SV_sample_ values were measured according to ISO 3657:^2013^, as previously described (ISO 2013; Ingram and Winterburn 2021).

### Analysis of oil samples

The crude SCG oil was previously analyzed for its composition (%, w/w) of organic elements (C, H, N, S, and O) and fatty acids (Ingram and Winterburn 2022). The saponified oil samples were analyzed using the same methods as previously described (Ingram and Winterburn 2021). Briefly, a Flash 2000 Organic Elemental Analyser (Thermo Fisher Scientific, UK) was used to determine the composition of C, H, N, and S. The quantitative analysis of K in the samples was performed via inductively couple plasma-optical emission spectroscopy (ICP-AES) using an iCAP 6300 Duo ICP spectrometer (Thermo Fisher Scientific, UK). The remaining fraction of the organic elements was assumed to be oxygen. Fatty acids were quantified with gas chromatography equipped with a flame ionization detector (GC-FID) after their conversion to fatty acid methyl esters via methanolysis.

### Fermentation media

*C. necator* DSM 545 was cultivated in defined mineral media (modified DSMZ medium 81) at 30 °C and pH 6.8–7.0 for a duration of 24–72 h. Crude and saponified SCG oils were supplied as the sole carbon sources at concentrations later described. NH_4_Cl was added as the sole nitrogen source at an initial concentration of 2.17 g/L. Other media components were present in the following concentrations (g/L): KH_2_PO_4_ (2.32), Na_2_HPO_4_⋅2H_2_O (2.93), MgSO_4_⋅7H_2_O (0.51), CaCl_2_⋅2H_2_O (0.010), MnCl_2_⋅4H_2_O (0.005), ferric ammonium citrate (0.050), and 5 mL trace element solution. The composition of the trace element solution (g/L) was as follows: ZnSO_4_⋅7H_2_O (0.10), MnCl_2_⋅4H_2_O (0.03), H_3_BO_3_ (0.30), CoCl_2_⋅6H_2_O (0.20), CuCl_2_⋅2H_2_O (0.01), NiCl_2_⋅6H_2_O (0.02), Na_2_MoO_4_⋅2H_2_O (0.03). The crude and saponified oils, phosphate solutions, and trace element solutions were prepared and autoclaved separately (121 °C, 25 min) and then reconstituted aseptically at room temperature. A concentrated stock saponified oil solution was prepared using the previously determined conditions (0.50 mol_KOH_/L, 50 °C, 90 min), subsequently dried to constant weight in a vacuum oven at 80 °C to remove the excess ethanol, and then dissolved in distilled water. 1 mol/L solutions of KOH and HCl were used to adjust the pH of the separate media solutions to 6.8–7.0 before autoclaving. Each fermentation was inoculated with 10% (v/v) of a preculture solution that was developed in shake flasks (30 °C and 200 rpm) containing rich media (DSMZ medium 1) over a 2-stage process, with each stage lasting 24 h (Ingram and Winterburn 2021) (Table 1).

**Table 1:** Substrate inhibition models used in this study

Model	Equation	References
Monod	$\mu=\frac{\mu_{max}C_0}{K_s+C_0}$	Monod (1949)
Andrews	$\mu=\frac{\mu_{max}{\mathrm C}_0}{\left({\mathrm K}_s+{\mathrm C}_0\right)\left(1+\frac{C_0}{{\mathrm K}_{\mathrm i}}\right)}$	Andrews (1968)
Aiba	$\mu=\frac{\mu_{max}{\mathrm C}_0}{{\mathrm K}_{\mathrm s}+{\mathrm C}_0}e^{\left(\frac{-{\mathrm C}_0}{{\mathrm K}_{\mathrm i}}\right)}$	Aiba et al. (1968)
Haldane	$\mu=\frac{\mu_{max}{\mathrm C}_0}{{\mathrm K}_{\mathrm s}+{\mathrm C}_0+\frac{{\mathrm C}_{max}}{{\mathrm K}_{\mathrm i}}}$	Haldane (1965)
Han-Levenspiel	$\mu=\frac{\mu_{max}C_0\left[1-\frac{C_0}{C_{max}}\right]^n}{C_0+{=K}_s\left[1-\frac{C_0}{C_{max}}\right]^m}$	Han and Levenspiel (1988)
Luong	$\mu=\frac{\mu_{max}{\mathrm C}_0}{{\mathrm K}_{\mathrm s}+{\mathrm C}_0}\left[1-\frac{{\mathrm C}_0}{{\mathrm C}_{max}}\right]^{\mathrm n}$	Luong (1987)
Moser	$\mu=\frac{\mu_{max}{\mathrm C}_0^{\mathrm n}}{{\mathrm K}_{\mathrm S}^{\mathrm n}+{\mathrm C}_0^{\mathrm n}}$	Moser (1958)
Webb	$\mu=\frac{\mu_{max}{\mathrm C}_0\left(1+\frac{{\mathrm C}_0}{\mathrm K}\right)}{{\mathrm K}_{\mathrm s}+{\mathrm C}_0+\left(\frac{{\mathrm C}_0^2}{{\mathrm K}_{\mathrm i}}\right)}$	Webb (1963)
Yano	$\mu=\frac{\mu_{max}{\mathrm C}_0}{{\mathrm K}_{\mathrm s}+{\mathrm C}_0+\frac{{\mathrm C}_0^2}{{\mathrm K}_{\mathrm i}}\left(1+\frac{{\mathrm C}_0}{\mathrm K}\right)}$	Yano et al. (1966)

*HL* Han-Levenspiel model

*Symbols:* *μ*, specific growth rate (h^−1^); *µ*_*max*_, maximum specific growth rate (h^−1^); *C*_0_, initial carbon substrate concentration (g/L); *K*, positive substrate dissociation constant (g/L); *K*_*i*_, inhibition constant (g/L); *K*_*s*_, half-saturation constant (g/L); *n* and *m*, constants

### Shake flask cultivations

Fermentations were initially carried out in 250-mL shake flasks containing 50 mL of DSMZ medium 81 to assess if saponification influences the ability of *C. necator* DSM 545 to consume the SCG oil in the first 24-h cultivation period. SCG oil was supplied as the sole carbon source at a range of initial concentrations of 3.72–30.00 g/L. For comparison, the conditions were repeated using saponified SCG oil (prepared with the optimized conditions), as the sole carbon source, at matching carbon concentrations of the crude SCG oil cultivations (Table 2). Hence, the initial C/N ratio (C/N_0_) ranged from 5.0 to 40.2 (g/g). Cultivation conditions were performed in duplicate. Triplicate samples were taken at the start (*t* = 0 h) and end (*t* = 24 h) of the cultivation period to determine the changes in the total biomass concentration, as measured by optical density at 600 nm, OD_600_.

**Table 2:** Composition of fatty acids and organic elements of crude and saponified SCG oils

Component	SCG Oil	
	Crude^a^	Saponified^b^
*Fatty acid (%, w/w)*		
Palmitic (C16:0)	31.4 ± 0.4	30.9 ± 0.3
Stearic (C18:0)	6.6 ± 0.1	6.6 ± 0.2
Oleic (C18:1)	17.1 ± 0.2	17.1 ± 0.2
Linoleic (C18:2)	39.6 ± 0.2	38.2 ± 0.3
α-Linolenic (C18:3)	2.5 ± 0.1	2.6 ± 0.1
Arachidic (C20:0)	0.6 ± 0.0	0.5 ± 0.1
Behenic (C22:0)	0.5 ± 0.0	0.5 ± 0.0
Other	1.7 ± 0.5	3.6 ± 0.5
*Elements (%, w/w)*		
C	76.74 ± 0.29	58.11 ± 1.38
H	11.66 ± 0.01	9.25 ± 0.28
N	n.d	n.d
S	n.d	n.d
K	n.d	12.92 ± 0.09
O_calculated_	11.61 ± 0.30	19.66 ± 1.75

^a^Oil was previously analyzed (Ingram and Winterburn 2022)

^b^Oil was saponified using 0.5 mol/L KOH in ethanol at 50 °C for 90 min

Saponifying the SCG oil causes the potassium concentration in the aqueous media to increase to levels that potentially inhibit cell growth. Therefore, additional fermentations were carried out in shake flasks containing DSMZ medium 81, supplemented with 5 g/L of glucose and different concentrations of KCl ([K] = 0–20 g/L).

### Estimation of kinetic growth model parameters

Nine Monod-based kinetic growth models (Table 1) were fitted to the shake flask cultivation data to evaluate the relationship between *μ* and *C*_0_. The parameters, function, and application of these models have been previously described in detail (Panikov 2010; Dutta et al. 2015; Muloiwa et al. 2020). Values of *μ* for the shake flask cultivations were calculated using Eq. (2).

2 $$\mu=\frac{\ln\;\left(\frac{{\mathrm{OD}}_{600,\;t=24\;\mathrm h}}{{\mathrm{OD}}_{600,\;t=0\;\mathrm h}}\right)}{24\;\mathrm h}$$

The parameters of each model were estimated using non-linear regression analysis performed with a bespoke Python (ver. 3.8.0) program applying SciPy “curve_fit” (ver. 1.5.2), as seen at https://github.com/ristojm/Biofit. The coefficient of determination (*R*^2^) and the root mean squared error (RMSE) are both error functions used to determine the robustness of a model. *R*^2^ measures the degree of linear correlation between the observed and predicted values. In general, a value of *R*^2^ closer to 1 indicates a more valid model. Residuals are the difference between the observed and predicted values, and they describe the concentration of the data around the regression line. Furthermore, RMSE is the standard deviation of the residuals and serves to combine the scale of the errors in predictions into a single measure of predictive power. Values of RSME closer to 0 indicate a closer fit to the data. Values of *R*^2^ and RMSE for each model were determined using Eqs. (3) and (4), respectively.

3 $$\mathrm R^2=1-\frac{\sum_{\mathrm i=1}^{\mathrm n}\left({\mathrm y}_{\mathrm i}-{\widehat{\mathrm y}}_{\mathrm i}\right)^2}{\sum_{i=1}^{\mathrm n}\left({\mathrm y}_{\mathrm i}-\overline{\mathrm y}\right)^2}$$

4 $$\mathrm{RMSE}=\sqrt{\frac{\sum_{\mathrm i=1}^{\mathrm n}\left({\mathrm y}_i-{\widehat{\mathrm y}}_{\mathrm i}\right)^2}{\mathrm n}}$$

where *i* and *n* are the index and upper limit of summation, respectively; and *y*, *ŷ*, and *ȳ* are observed, predicted, and mean of the observed values of *µ*, respectively.

### Bioreactor cultivations

Bioreactor cultivations were performed as previously described (Ingram and Winterburn 2021) based on the optimal conditions surmised from the shake flask cultivation and accompanying analysis of the growth kinetics. Briefly, 3-L bioreactors (Applikon Biotechnology, UK) were operated in batch mode with a 2.2-L working volume (200 mL of the 2nd-stage preculture inoculated into 2 L of fresh mineral salt media). In the first batch experiment, crude SCG oil was initially supplied as the sole carbon source at 12.47 g/L (*C*_0_ = 9.57 g/L). Another batch cultivation was carried out in which a mixture of crude and saponified SCG oils was supplied at the same *C*_0_, but with a C-ratio between the crude and saponified oils of 75:25% (w/w). Air was supplied at 1.0 vvm, with dissolved oxygen (DO) maintained at 30% saturation level through variation of the rate of agitation (600–800 rpm). Process temperature was maintained at 30 ± 0.2 °C. pH was controlled 7.0 ± 0.1 via pulse additions of 3 mol/L solutions of HCl/NaOH. Propylene glycol, fed into the bioreactor via a pump connected to a level sensor, was used to suppress any foaming.

### Analytical methods

Bioreactor samples taken for the determination of optical density at 600 nm (OD_600_), total biomass (g/L), and PHA (g/L) were prepared and analyzed as previously described (Ingram and Winterburn 2021). The cells were washed with n-hexane to remove any residual oils before analysis. Residual biomass was defined as the difference between the total biomass and PHA concentrations. For total nitrogen (TN), samples were initially prepared by taking 0.1 mL aliquots from the cell-free supernatants of the OD_600_ samples, diluted to a final volume of 20 ml with HPLC grade-water, and filtered through 0.45-μm pore nylon syringe filters into sample vials (Fisher Scientific, UK). Analysis was performed using a TOC-VCPH Total Organic Carbon analyzer, coupled with a TNM-1 TN analyzer unit (Shimadzu, UK), as previously described (Urbina et al. 2018; Wongsirichot 2020). Standards for the TN were made from NH_4_Cl.

### Calculations

PHA accumulation, biomass, and PHA yield coefficients (with respect to initial oil, carbon and nitrogen concentrations) and volumetric productivities for each fermentation condition were all calculated as previously described (Ingram and Winterburn 2021).

## Results

### Saponification of SCG oil

**Fig. 1:**
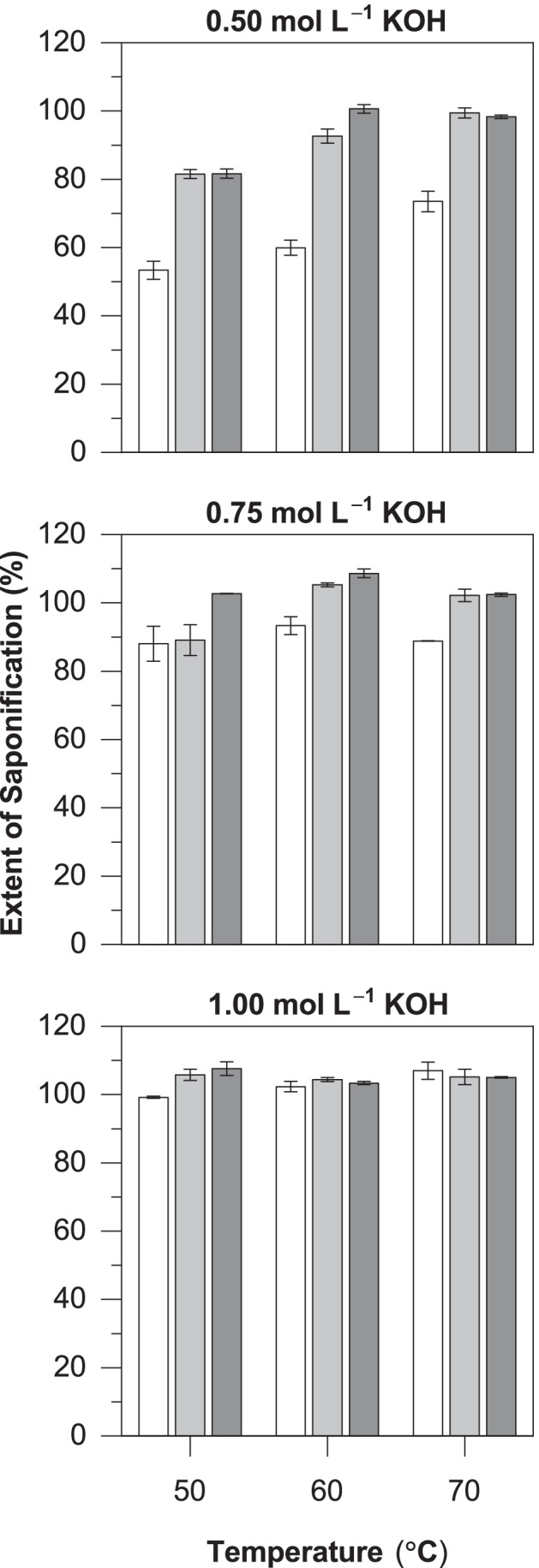
Extent of saponification (%) of SCG oil at different KOH concentrations, temperatures, and reaction times (30 min *white bars*, 60 min *light-gray bars*, and 90 min *dark-gray bars*)

The saponification of SCG oil was carried out at different KOH concentrations (0.5–1.0 mol/L) and temperatures (50–70 °C) for varying lengths of time (30–90 min) to find the mildest conditions able to liberate the constituent fatty acids completely, as defined by the extent of saponification (%). The results of this process are summarized in Fig. 1. The mildest evaluated conditions (0.50 mol_KOH_/L, 50 °C, 30 min) achieved the lowest extent of saponification, at 53.4 ± 2.6%. Increasing any of the three variables increased the overall conversion. At 90 min, conversions of ≥ 98% were achieved with all evaluated combinations of temperature and KOH concentration, except for the mildest condition (0.50 mol_KOH_/L, 50 °C), which reached 81.7 ± 1.4%. Using 1.00 mol_KOH_/L resulted in complete saponification, regardless of which temperature or time was used. Hence, the conditions selected to saponify the oil in subsequent fermentation experiments were 0.50 mol_KOH_/L, 50 °C, and 90 min.

### Analysis of crude and saponified SCG oil

The crude SGC oil was previously analyzed in terms of its elemental and fatty acid compositions and acid and saponification values (Ingram and Winterburn 2022). The same analyses were performed on the oil samples saponified using the previously established conditions (0.50 mol_KOH_/L, 50 °C, 90 min). The results of these analyses are displayed in Table 2.

### Effect of potassium on cell growth

Potassium-induced inhibition of *C. necator* DSM 545 was evaluated by cultivating the cells in shake flasks supplemented with different concentrations of KCl (0–38.1 g/L ([K] = 0–20 g/L)) and monitoring the change in OD_600_ after 24 h. All cultivations started with an initial OD_600_ value of 0.24. Cells grown in the absence of supplemented potassium reached an OD_600_ value of 2.1 ± 0.0. In comparison, OD_600_ increased to 2.3 ± 0.1 at [K] = 2.5, 5.0 or 10.0 g/L. However, cell growth was inhibited at [K] = 20.0 g/L, as OD_600_ reached only 0.9 ± 0.0.

### Effect of SCG oil saponification on cell growth

**Fig. 2:**
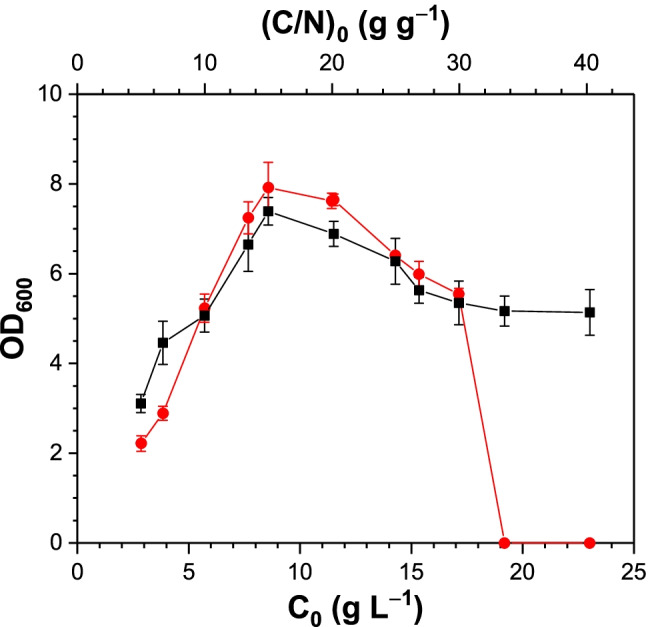
Values of OD_600_ obtained for shake flask cultivations of *C. necator* DSM 545 grown on crude (*black squares*) and saponified (*red circles*) SCG oils for 24 h, at initial carbon concentrations, *C*_0_, of 2.9–23.0 g/L. Also shown are the corresponding initial carbon–nitrogen ratios, (C/N)_0_

*C. necator* DSM 545 was cultivated in shake flasks supplemented with either crude or saponified SCG oil as the sole carbon source (*C*_0_ = 2.9–23.0 g/L) to evaluate the effects of saponification on the organism’s ability to consume the substrate. All cultivations started with an initial OD_600_ value of 0.24. Figure 2 shows the values of OD_600_ obtained after 24 h. The effect of *C*_0_ on the OD_600_ values largely followed the same pattern for both substrates. For crude SCG oil, values of OD_600_ steadily rose with increasing *C*_0_, rising from 3.1 ± 0.2 (*C*_0_ = 2.9 g/L) to a peak of 7.4 ± 0.3 (*C*_0_ = 8.6 g/L). At the same concentrations, values of OD_600_ for cells grown on saponified SCG oil rose from 2.2 ± 0.2 to a higher peak of 7.9 ± 0.6. Further increases of *C*_0_ caused the resulting OD_600_ to decline for both substrates. Once the *C*_0_ reached 19.2 g/L, cell growth was entirely inhibited in the saponified SCG oil flasks, as no biomass could be detected. Similar results were reported by Bhatia et al. (2018) during their cultivations of *C. necator* Re2133 on SCG oil to synthesize poly(3-hydroxybutyrate-*co*-3-hydroxyhexanoate) (P(3HB-*co*-3HHx)) copolymers. The authors observed biomass concentrations peaked at a C/N_0_ ratio of 20 g/g. In the present study, the peak OD_600_ values obtained at *C*_0_ = 8.6 g/L equated to an initial C/N_0_ ratio of 15.0 g/g.

### Substrate inhibition growth kinetics

**Fig. 3:**
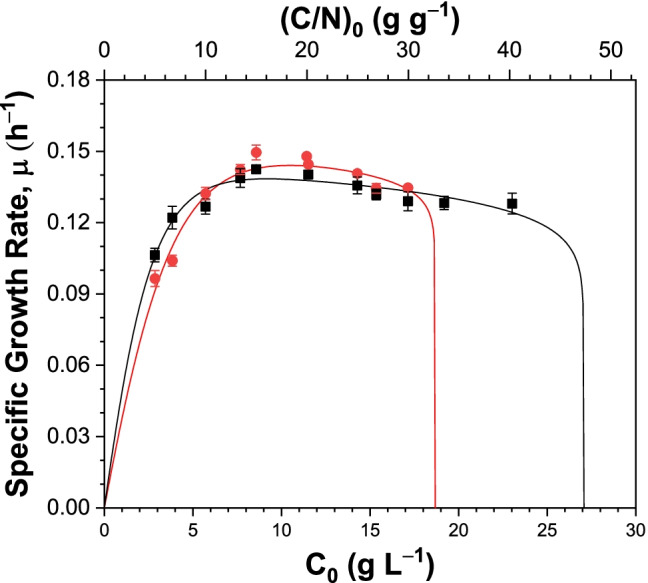
Comparison of *µ* (h.^−1^) values calculated experimentally (*symbols*) and from the Han-Levenspiel model (*lines*) for shake flask cultivations of *C. necator* DSM 545 grown on crude (*black squares/line*) and saponified (*red circles/line*) SCG oils for 24 h, at initial carbon concentrations, *C*_0_, of 2.9–23.0 g/L. Also shown are the corresponding initial carbon–nitrogen ratios, (C/N)_0_

The relationship between *μ* and *C*_0_ was assessed by fitting the kinetic growth models (Table 1) to the results of the shake flask cultivations. The model parameters, estimated via non-linear regression, are shown in Table 3. All the models predicted the organism to have maximum specific growth rates, *μ*_max_, ranging between 0.140–0.145 and 0.147–0.152 when grown on the crude and saponified SCG oils, respectively. The Han-Levenspiel model had the closest fit to the experimental data for both substrates (Fig. 3), as indicated by the *R*^2^ and RMSE values. Additionally, the model correctly predicted that cell growth would be completely inhibited at *C*_0_ values higher than the critical substrate concentration (*C*_max_) of 19.1 g/L for the saponified oil. Hence, the Han-Levenspiel model is the most suitable to describe the specific growth rate of the organism under these fermentation conditions. According to the model, peak *µ*-values of 0.139 h^−1^ and 0.145 h^−1^ were reached with the crude and saponified SCG oil concentrations of 11.99 g/L (*C*_0_ = 9.20 g/L) and 17.40 g/L (*C*_0_ = 9.94 g/L), respectively. The mid-point of these two values (*C*_0_ = 9.57 g/L) was used in subsequent cultivations.

**Table 3:** Estimated parameters of the substrate inhibition models

Model	Estimated parameters							*R*^2^	RMSE
	*µ*_max_	*K*_*s*_	*K*_*i*_	*C*_max_	*n*	*m*	*K* (g/L)		
	(h^−1^)	(g/L)	(g/L)	(g/L)					
Crude SCG oil									
Monod	0.144	0.69	−	−	−	−	−	0.398	0.007
Aiba	0.144	0.61	731.08	−	−	−	−	0.511	0.007
Andrews	0.144	0.61	736.03	−	−	−	−	0.509	0.008
Haldane	0.144	0.68	35.69	0.35	−	−	−	0.398	0.007
HL	0.145	2.65	−	28.50	0.096	8.995	−	0.885	0.003
Luong	0.141	0.61	−	23.25	2.091	−	−	0.439	0.013
Moser	0.144	0.61	−	−	0.021	−	−	0.628	0.006
Webb	0.144	3.84	0.69	−	−	−	0.72	0.575	0.006
Yano	0.144	0.60	6.43E + 07	−	−	−	1.13E-04	0.540	0.006
Saponified SCG oil									
Monod	0.151	1.06	−	−	−	−	−	0.670	0.060
Aiba	0.151	1.03	7530.31	−	−	−	−	0.663	0.059
Andrews	0.151	1.03	7582.16	−	−	−	−	0.663	0.059
Haldane	0.151	1.06	88.14	0.51	−	−	−	0.670	0.060
HL	0.152	4.89	** − **	19.11	0.058	5.456	−	0.947	0.004
Luong	0.151	0.99	−	17.14	0.003	−	−	0.671	0.009
Moser	0.149	0.58	−	−	1.385	−	−	0.759	0.062
Webb	0.151	3.84	1.49	−	−	−	1.57	0.799	0.059
Yano	0.151	0.95	1.13E + 07	−	−	−	2.19E-03	0.639	0.059

*RMSE* root mean square errors, *HL* Han-Levenspiel model

### Saponified SCG oil as a surfactant

A final set of shake flask cultivations was carried out in which crude and saponified SCG oils were mixed in incremental ratios to evaluate the resulting effects on cell growth; the results are shown in Table 4. By replacing 25% (w/w) of the initial carbon supplied from the crude SCG oil with the saponified substrate, the resulting OD_600_ value increased by 20.1%, from 7.8 ± 0.2 to 9.4 ± 0.1. Further substitution of the crude oil resulted in a slow decline of the OD_600_ values.

**Table 4:** Values of OD_600_ obtained for shake flask cultivations of *C. necator* DSM 545 grown on mixtures of crude and saponified SCG oils in varying carbon–carbon ratios (*C*_0_ = 9.57 g/L) for 24 h

Initial carbon ratio (%, w/w)		OD_600_
Crude SCG oil	Saponified SCG oil	
100	0	7.8 ± 0.2
75	25	9.4 ± 0.2
50	50	9.0 ± 0.2
25	75	8.4 ± 0.1
0	100	7.1 ± 0.1

### Bioreactor fermentations

**Fig. 4:**
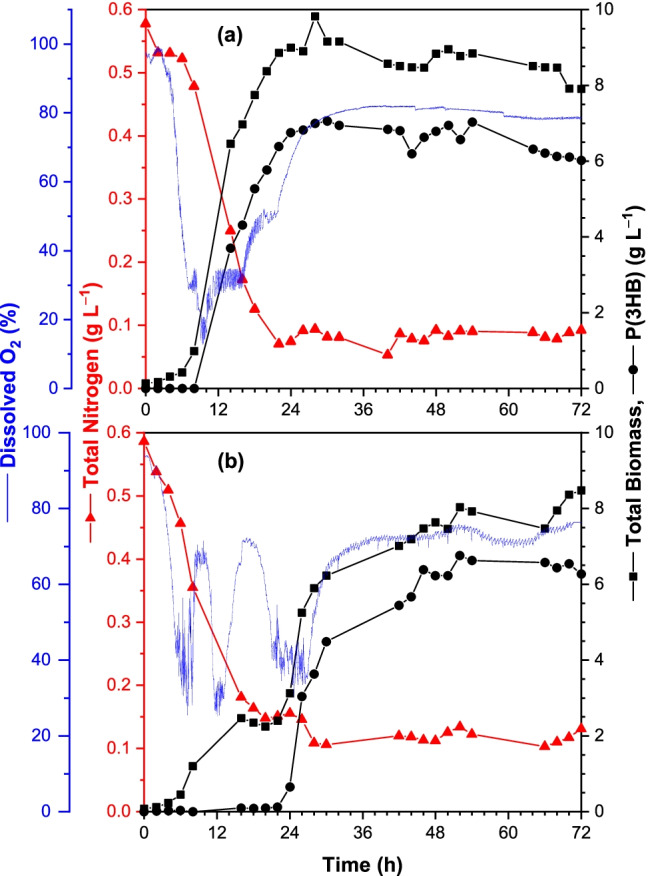
Time course of substrate consumption and biomass and PHA production during the cultivations of *C.* *necator* DSM 545 grown on **a** 12.47 g/L crude SCG oil; and **b** 9.35 g/L crude SCG oil and 4.12 g/L saponified SCG oil. In both conditions, *C*_0_ = 9.57 g/L

*C. necator* DSM 545 was then cultivated in 3-L bioreactors (batch) for 72 h to evaluate if saponifying a portion (25% C, w/w) of the SCG oil helps to improve the overall fermentation process by emulsifying the remaining crude fraction to increase its bioavailability. Fermentations were also carried out with 100% crude SCG oil for comparison. Figure 4 shows the progression of substrate (N and DO) consumption and biomass (total and PHA) production.

Cells grown on only crude SCG oil experienced an initial lag phase (0–2 h) as they adapted to the new environmental conditions, synthesizing extracellular lipases to hydrolyze the acylglycerides present in the crude SCG oil (*C*_0_ = 9.57 g/L), forming free fatty acids and glycerol (Budde et al. 2011; Lu et al. 2013). The subsequent exponential growth phase (2–8 h) was characterized by a *µ*-value of 0.23 h^−1^. During the next unmeasured period (8–14 h), an essential nutrient for growth, such as P, K, or Mg, became depleted (neither N nor O were depleted as indicated by their measured values) as carbon remained in excess. In response, the cells adapted their metabolism to funnel the available carbon into P(3HB) production. Details of how *C. necator* shifts its metabolism from supporting cellular growth to PHA production has been described elsewhere in detail (Kessler and Witholt 2002; Ray and Kalia 2017; Ingram and Winterburn 2021). At this point, the total biomass concentration ([TB]) rapidly increased from 1.0 g/L (*t* = 8 h) to a peak value of 9.8 g/L at 28 h, plateauing thereafter. The corresponding concentration of P(3HB) at this peak was 7.0 g/L, equating to 71.4% (w/w) of the total biomass.

In the cultures employing the crude and saponified SCG oil mixture, the cells exhibited two distinct growth phases somewhat akin to diauxic growth, and a final PHA production period. Initially (0–8 h), cells consumed the saponified substrate ([*C*] = 2.39 g/L) for cellular growth without an observed initial lag phase, reaching a [TB] value of 1.2 g/L. The specific growth rate (*µ*) during this period was 0.35 h^−1^. This was followed by a lag phase (8–11 h, based on the O_2_ consumption profile) and a secondary growth phase (11–13 h) to reach a [TB] value of 2.4 g/L. After a second lag phase (13–22 h), the cells synthesized and accumulated 3HB monomers from the available carbon in the form of FFAs (22–72 h). The highest P(3HB) concentration of 6.8 g/L was reached at 52 h, corresponding to 84.2% (w/w) of the total biomass (8.0 g/L). Nitrogen was steadily consumed over the entire cellular growth phase, dropping from 0.59 to 0.13 g/L by the start of the PHA production phase and plateauing after that.

Polymer containing 3-hydroxyvalerate (3HV) was detected at the start in both cultivations and remained present throughout at concentrations in the range of 0.04–0.37 g/L. The precultures were developed from seed stocks stored in cryo-storage solutions containing 20% (v/v) glycerol which could be metabolized in such a way that led to the formation of 3HV-containing polymers (Ingram and Winterburn 2021, 2022). *C. necator* DSM 545 has been observed to produce 3HV distinctly after producing 3HB once the usable even-chained fatty acids in plant oils had been consumed (Ingram and Winterburn 2021). However, no such phase was observed in the present study (data not shown).

Table 5 compares the overall fermentation performances of both substrates with respect to the biomass (total and PHA) concentrations, yields, and volumetric productivities. The presented data is reflective of the time at which the fermentation productivity effectively stops. Cultures grown on the mixed substrates produced slightly more PHA (6.8 g/L) than those grown on just the SCG oil (6.6 g/L), but took over twice as long to do so; the volumetric productivities are reflective of that. The PHA yields are similar for both cultures.

**Table 5:** Comparison of biomass and P(3HB) production of *C. necator* DSM 545 cultivated on crude and saponified SCG oils in 3 L bioreactors (batch)

Substrate	Crude^a^	Cru./Sap.^b^
Analyzed point (h)^c^	24	52
Feedstock (g/L)		
Crude SCG oil	12.47	9.35
Saponified SCG oil	–	4.12
C	9.57	9.57
N	0.57	0.57
C/N (g/g)	16.8	16.8
Biomass and PHA (g/L)		
Total biomass	9.0 ± 0.2	8.0 ± 0.2
P(3HB)	6.6 ± 0.4	6.8 ± 0.2
P(3HB) (%, w/w)	73.2 ± 1.9	84.2 ± 1.1
Residual biomass	2.4 ± 0.4	1.3 ± 0.3
Biomass yields (g/g)		
* Y*_x/oil_	0.72	0.64^d^
* Y*_x/C_	0.94	0.84
* Y*_x/N_	15.79	14.04
PHA yields (g/g)		
* Y*_PHA/oil_	0.53	0.54 ^d^
* Y*_PHA/C_	0.69	0.71
* Y*_PHA/N_	11.58	11.92
Volumetric productivity (g/L/h)		
Total biomass	0.38	0.15
PHA	0.28	0.13

^a^100% crude SCG oil

^b^Blend (75:25% C, w/w) of crude and saponified SCG oil

^c^Time at which cultivation effectively finished

^d^Calculated with respect to crude SCG oil equivalent

## Discussion

The relatively low cost and high abundance of waste plant oils make them attractive carbon substrates in PHA production. The immiscibility between plant oil substrates and aqueous fermentation media causes several issues, the chief among which is the induced limitation on substrate bioavailability. Surfactants can be used to form emulsified oil-in-water solutions to overcome this issue; this strategy has been successfully employed in highly reproducible bioreactor cultivations *C. necator* H16 to shorten the initial lag phase (Budde et al. 2011). However, surfactants are expensive and are thus unlikely to be used in the industrial production of PHAs where production costs remain high (Budde et al. 2011; Riedel et al. 2012; Surendran et al. 2020; Ingram and Winterburn 2021). The present study sought a similar solution, but via saponification of the oil instead. Optimizing the saponification reaction conditions (Fig. 1) is necessary to minimize the resources required in the additional processing step, which would otherwise add to the overall PHA production cost. Total saponification was achieved with relatively mild conditions (0.50 mol_KOH_/L, 50 °C, 90 min), suggesting the saponification process could be further optimized. The saponification value of the SCG oil used in the current study was previously determined to be 175.7 ± 2.3 mg_KOH_ goil^−1^ (Ingram and Winterburn 2022), meaning the lowest KOH concentration able to saponify the SCG oil under the proposed conditions entirely is 0.313 mol/L.

Using the selected saponification conditions, potassium comprised 12.92 ± 0.09% (w/w) of the saponified oil (Table 2). This figure is essential to consider when adding the substrate to the fermentation process, as cell growth is inhibited at [K] = 20.0 g/L. To introduce that much potassium would require the addition of 155 g/L of saponified SCG oil, which is substantially more than what was used in the present study (6.6–39.6 g/L). However, higher concentrations could easily be considered in the pursuit of greater PHA productivities, especially in fed-batch of continuous type fermentations. Sucrose, which has a C-fraction of 44.11% (w/w), has been previously used at a concentration of 900 g/L in a fed-batch fermentation of *Alcaligenes latus* DSM 1123 to produce the highest ever reported PHA volumetric productivity at 5.13 g/L h^−1^ (Wang and Lee 1997; Blunt et al. 2018). To supply the equivalent amount of carbon would require 683 g/L of saponified SCG oil, which would add 88.2 g/L of potassium. Thus, further improvements to the initial saponification step to lower the amount of potassium introduced would help to increase the maximum amount of substrate that could be supplied.

To improve the biopolymer yield and volumetric productivities from SCG oil, the present study experimentally evaluated the relationship between *C*_0_ and *µ*, and subsequently, nine kinetic growth models (Table 1) were curve-fitted to the results via non-linear regression analysis. Increasing the initial substrate concentration resulted in progressively higher cell densities for both crude and saponified SCG oils, but further increases began to inhibit cell growth (Fig. 2). Among the evaluated models, the Han-Levenspiel model resulted in the best curve fit to the experimental values (Fig. 3), with *R*^2^ and RMSE values of 0.885 and 0.003 for the crude SCG oil, and 0.947 and 0.004 for the saponified SCG oil, respectively (Table 3). This model extends the Monod model, accounting for inhibitory effects associated with cell, product, and substrate concentrations. The model also predicts cell growth will cease entirely at a critical inhibitory concentration of the substrate, *C*_max_, and describes the type of inhibition (competitive, non-competitive or uncompetitive) based on the values of the two constant parameters, *n* and *m* (Han and Levenspiel 1988; Dutta et al. 2015). The model predicted that *C*_max_ would be 28.5 and 19.1 g/L (initial carbon concentration) for the crude and saponified oils, respectively. The latter was experimentally observed (Figs. 2 and 3). Based on the values of *n* and *m* (Table 3), the model suggests that the inhibition is uncompetitive (*m* > *n* > 0) for both substrates. Understanding the relationship between *C*_0_ and *µ* provides a rationale for designing suitable substrate feeding regimes. The subsequent shake flask cultivations—to determine the influence of mixing the crude and saponified SCG oils—were carried out at the optimal *C*_0_ value determined from the prior experimental modeling: *C*_0_ = 9.57 g/L.

Artificially increasing the FFA content of SCG oil—achieved by increasing the fraction of saponified oil in the mixtures—was confirmed in the present study to improve the bioavailability of the substrate (Table 4) up to a certain point, beyond which further substitutions of the crude SCG oil negatively influenced cell growth. The FFA content of plant oils has been shown to positively impact the growth and PHA production of *C. necator* as FFAs act to emulsify the remaining constituent triacylglycerides, making them more accessible to the action of lipases (Obruca et al. 2014). This influence would likely be more pronounced in other plant oils, which typically have much lower acid values than SCG oils (Al-Hamamre et al. 2012; Obruca et al. 2014; Ingram and Winterburn 2022). The option to effectively control the FFA content through altering the ratio of crude to saponified SCG oil is a powerful tool to allow the user to influence how the substrate ultimately behaves in the aqueous environment.

The findings of the shake flask experiments culminated in the final set of bioreactor cultivations. *C. necator* 545 was grown on a blend (75:25% C, w/w) of crude and saponified SCG oil (*C*_0_ = 9.57 g/L), benchmarked against 100% crude SCG oil at the same *C*_0_ (Fig. 4). The cells grew 1.52 times faster on the mixed substrates during the first exponential growth phase (*µ* = 0.35 h^−1^) than on just crude SCG oil (*µ* = 0.23 h^−1^). However, after 8 h, the saponified oil substrate was depleted, and the cells had to adapt to consuming the non-saponified lipids (triacylglycerides). This lag phase had the unfortunate effect of delaying the start of PHA production by approximately 12 h compared to the crude oil cultures. The ultimate effect of this is that cells grown on the mixed substrates took around twice as long (52 h) to produce a similar level of PHA (6.8 g/L) than those grown on just the crude SCG oil (6.6 g/L in 24 h). By comparison, these values remain significantly lower than authors who cultivated the organism’s wild-strain (*C. necator* H16) on SCG oil (13.1–49.4 g/L) (Cruz et al. 2014; Obruca et al. 2014).

Employing a feeding strategy whereby pulsed additions of saponified SCG oil are fed into the bioreactor at regular intervals could help to capitalize on the initial faster cell growth to improve the overall efficiency of PHA production from the mixed substrates. A fed-batch feeding strategy was previously demonstrated to greatly enhance P(3HB) production by *C. necator* H16 from 26.5 g/L (batch: 30 g/L SCG oil) to 49.4 g/L (fed-batch: 60 g/L SCG oil) in a shorter period (Obruca et al. 2014). As the organism begins to produce PHAs after an essential nutrient (e.g., N, P, K, O, and Mg) reaches a limiting concentration (Ray and Kalia 2017), the maximum PHA concentration is predetermined by the number of cells produced during the initial growth phase. So, the aim is to achieve a high-enough cell density in as little time as possible. The pulses should be frequent enough and of sufficient quantity to avoid total depletion of the saponified SCG oil, but also restricted as to prevent substrate-induced inhibition, as well as the potential foaming effects.

More so than other plant oils, SCG oil causes foaming when used as the sole carbon source for the biosynthesis of PHAs in a bioreactor (Obruca et al. 2014). This effect is greatly exasperated when the oil is entirely saponified, as observed in the present study when cultivations utilizing 100% saponified SCG oil was also attempted (data not shown). Foaming is a major issue because it can lead to a loss of cells, reduces the effective volume of the bioreactor, and increases the risk overflow and contamination (Li et al. 1995). Although foaming was suppressed in the present study through the addition of polypropylene glycol, the use of chemical antifoaming agents can interfere with the oil extraction process by artificially increasing the measured lipid concentration (Budde et al. 2011). While non-metabolisable antifoams, such as polyglycols and silicone oils, have little effect metabolism of the cells, they can cause a reduction in the oxygen transfer efficiency, as well as membrane fouling (Delvigne and Lecomte 2010). These issues could potentially be avoided through the use of other plant oils, such as rapeseed oil, as antifoaming agents (Obruca et al. 2014), or by mechanical means (e.g., foam breakers or foam centrifuges) (Budde et al. 2011; Riedel et al. 2012).

Overall, plant oil saponification presents an exciting opportunity for overcoming their immiscibility in aqueous cultures, which has long been a bottleneck in PHA biosynthesis. *C. necator* DSM 545 was successfully cultivated for the first time on saponified SCG oil, achieving a total biomass concentration of 8.0 g/L after 52 h, with a high PHA content of 84.2% (w/w). Further, the relationship between *µ* and *C*_0_ was accurately characterized by the Han-Levenspiel model, which provided insights into the optimal starting substrate concentration, as well as the potential inhibitory limits. Though the cells grew significantly (52%) faster on the mixed substrates during the first exponential growth phase (*µ* = 0.35 h^−1^) than on just crude SCG oil (*µ* = 0.23 h^−1^), additional work is required to capitalize on this for improved PHA volumetric productivity.

## Data Availability

Not applicable.

## Code availability

The bespoke Python (ver. 3.8.0) program, which applied the SciPy “curve_fit” function (ver. 1.5.2) to estimate the parameters of the kinetic growth models, is available at https://github.com/ristojm/Biofit.
